# Supplementary figures and images for: A Refined Approach to Permanent Coronary Artery Ligation in Rats: Enhancing Outcomes and Reducing Animal Burden
Source: Animals (Basel). 2025 Dec 29;16(1):99. doi: 10.3390/ani16010099 (PMC12784918; doi:10.3390/ani16010099)

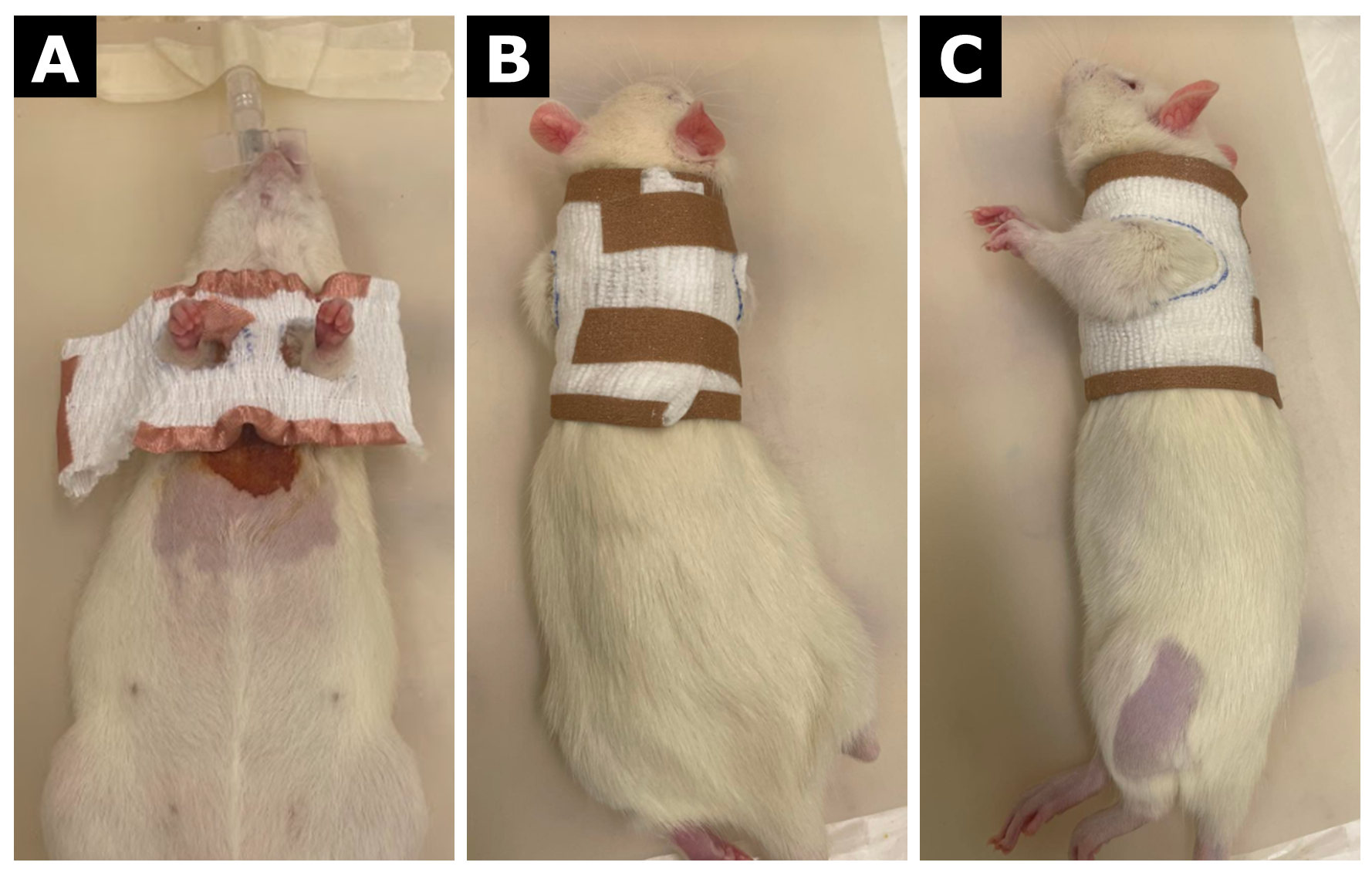

Supplement: Supplementary file 1 [file animals-16-00099-s001.zip › Figure S1_MI paper.tif]
